# Planting the seed: how parental negative conditional regard boosts vulnerability to excessive study behaviour and burnout

**DOI:** 10.1186/s40359-025-03354-z

**Published:** 2025-08-28

**Authors:** Malin Brueckmann, Elke Wild, Fabian Wolff

**Affiliations:** Department of Psychology, Bielefeld University, Universitätsstraße. 25, 33615 Bielefeld, Germany

**Keywords:** Academic burnout, Excessive study behaviour, Parental academic negative conditional regard, Self-esteem contingency

## Abstract

**Background:**

Excessive study behaviour as a precursor to academic burnout is receiving increasing attention in the research landscape. However, potential risk factors for this behaviour remain largely unconsidered. Against this background, this study, based on the self-esteem model of burnout, examines the risk-increasing influence of academic self-esteem contingency on burnout and extends the empirical research on this topic by investigating the mediating effect of excessive study behaviour. Moreover, it investigates the indirect effect of parental academic negative conditional regard at school age on burnout during university.

**Methods:**

A serial mediation model was used to analyse the research questions cross-sectionally across a sample of 624 students at German universities. The data was collected in the winter semester 2023/24.

**Results:**

Excessive study behaviour mediated the relationships between academic self-esteem contingency and the burnout dimensions of exhaustion and reduced self-efficacy. Moreover, parental academic negative conditional regard experienced during school age showed indirect effects on exhaustion and cynicism at university.

**Conclusion:**

The findings provide empirical support for the self-esteem model of burnout, which posits that burnout occurs as a result of a self-esteem that is contingent upon academic performance and compensatory excessive engagement. Furthermore, this study provides evidence of the long-term negative effects of parental academic negative conditional regard from school through the university years.

**Supplementary Information:**

The online version contains supplementary material available at 10.1186/s40359-025-03354-z.

## Introduction

The concepts of excessive study behaviour and academic burnout are particularly prominent in Western meritocracies and the subject of intensive international research, not least because of their high prevalence (6–17% for excessive study behaviour [1, 2] and up to 28% for burnout during university [3]). As with academic burnout, excessive study behaviour was initially investigated primarily in the context of the workplace, before the research was extended to the university context (e.g., [2,4]). The transfer of these constructs to the university context is based on clear parallels, including pre-structured tasks, planned submissions, and the further development of individual skills (cf., [5]). However, some students invest an excessive amount of effort in their studies and related tasks, to the extent that they neglect leisure activities and develop symptoms of burnout long before they even start working [2, 6–9].

Students suffering from academic burnout often perceive courses as a burden (exhaustion), feel unable to meet the demands of their studies (reduced self-efficacy), and ultimately lose interest in their field of study (cynicism; [10,11]). Academic burnout is therefore not only a significant health impairment for students, but also associated with other negative outcomes, including poorer academic performance [12] and a higher risk of dropping out [13, 14]. While the negative effects of excessive study behaviour in terms of predicting burnout and other long-term health impairments have already been well documented [2, 15–17], the investigation of predictors of such behaviour (not only in Germany) represents a research desideratum.

The high correlations between excessive study behaviour and academic burnout [6, 18] indicate a common origin. According to the self-esteem model of burnout [19], which assigns a high (predictive) value to academic self-esteem contingency in the process of burning out, self-esteem dependent on academic performance creates a vulnerability to excessive compensatory engagement and thus promotes emotional exhaustion. Associations between self-esteem contingency and individual burnout symptoms (exhaustion, cynicism, and reduced self-efficacy) have already been demonstrated in the university context [20]. However, there is a paucity of empirical evidence to support the mediation of the relationship through excessive study behaviour. Consequently, this mediation is examined as part of the first research question of this study.

**Fig. 1:**
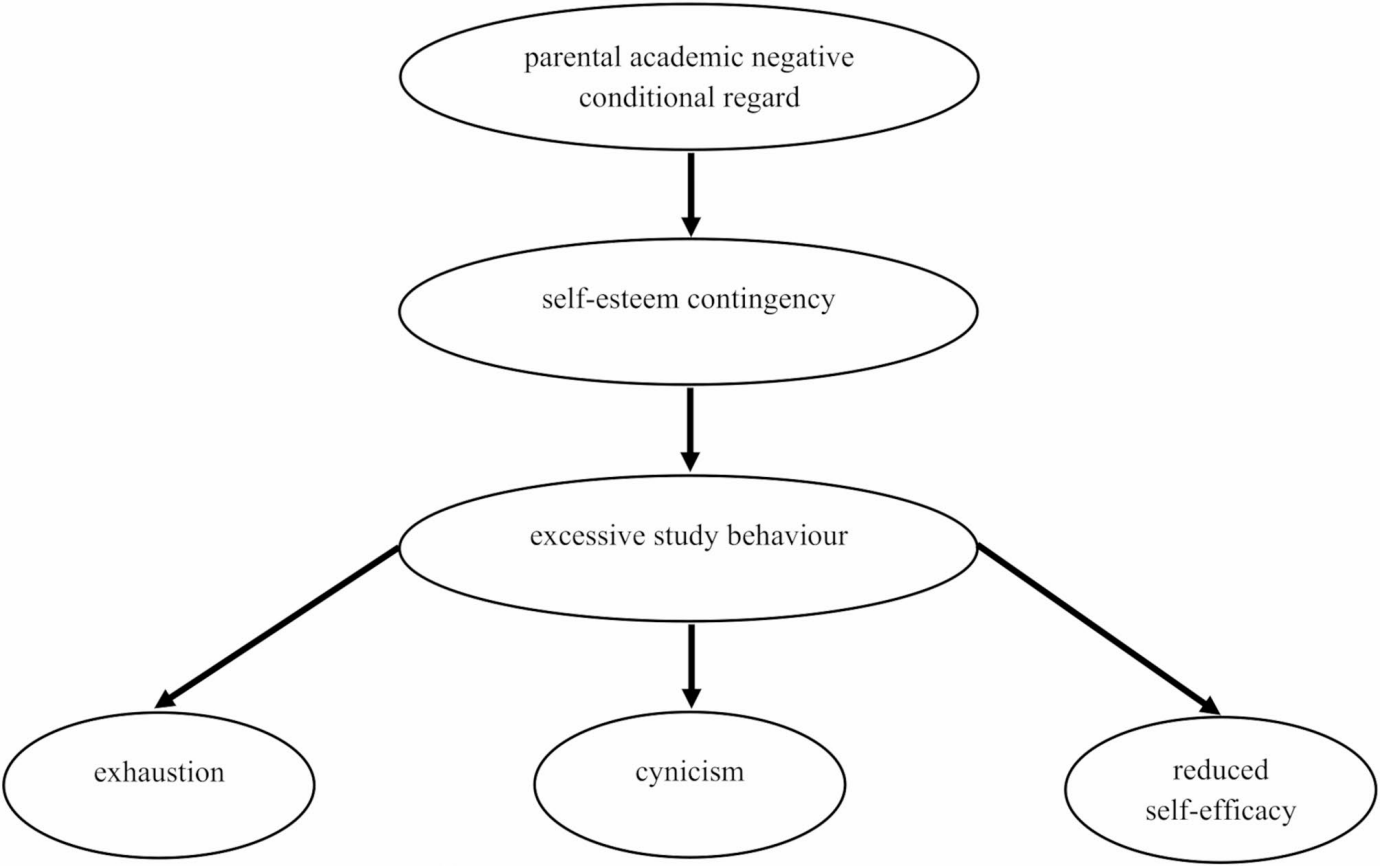
Theoretical Model

Furthermore, recent findings highlighted the importance of familial protective and risk factors in the development of academic burnout, particularly in school settings (e.g., [21,22]), and they identified parental academic (negative) conditional regard as a significant risk factor for the development of a self-esteem contingent on academic achievement (for an overview, see [23]). While the long-term effects of such parenting practices in later academic (e.g. university) or professional contexts have (to the best of our knowledge) not yet been systematically examined, it seems plausible to assume that parental academic negative conditional regard may continue to exert an influence beyond adolescence. Specifically, by fostering self-esteem that is closely linked to (academic) performance and considered a relatively stable personality trait, such relational experiences may indirectly increase the risk of maladaptive (i.e. excessive) study behaviour and burnout in emerging adulthood. This perspective helps to bridge the gap between developmental and educational psychology by highlighting the long-term processes underlying the development of burnout (e.g. self-esteem compensation via excessive study engagement). Therefore, a second research question examines the risk-increasing influence of parental academic negative conditional regard at school age on the development of burnout symptoms in the university context (serially mediated by self-esteem contingency and excessive study behaviour). Figure 1 illustrates the model to be tested and its hypothesised mechanisms of action, which are explained in the following sections.

**Table 1:** Latent means, standard deviations, and correlations

	Variable	M (SD)	1	2	3	4	5	6	7	8	9
1	parental negative conditional regard	1.82 (0.92)	1	0.23***	0.17***	0.13**	0.10*	0.09*	− 0.01	0.00	0.00
2	self-esteem contingency	2.97 (1.03)		1	0.56***	0.42***	0.19***	0.45***	0.00	0.26***	− 0.27***
3	excessive study behaviour	2.66 (0.80)			1	0.54***	0.12*	0.34***	0.06	0.24***	− 0.24***
4	exhaustion	2.91 (0.89)				1	0.67***	0.46***	− 0.61***	0.13**	− 0.13**
5	cynicism	2.23 (0.99)					1	0.66***	− 0.64***	− 0.04	0.04
6	reduced self-efficacy	2.58 (0.83)						1	− 0.52***	0.08	− 0.09*
7	academic engagement	2.97 (0.72)							1	0.00	0.00
8	female gender									1	− 0.97***
9	male gender										1

Note. * p < .05. ** p < .01. *** p < .001

**Fig. 2:**
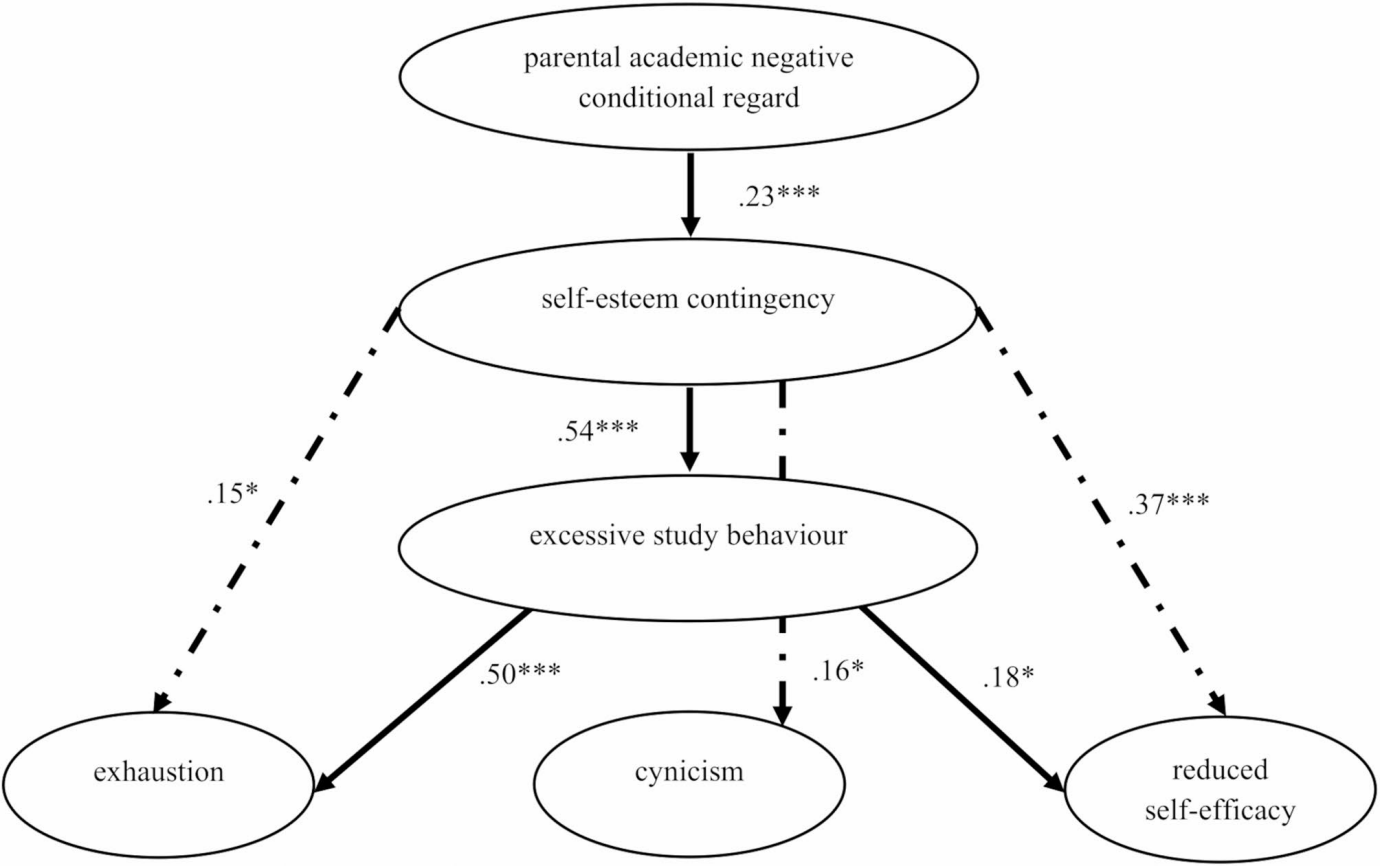
Model with All Significant Direct Paths *Note.* The path model was controlled for the effects of academic engagement and gender. Appendix 1 contains the full results. * *p* < .05. ** *p* < .01. *** *p* < .001

### Academic self-esteem as a key vulnerability factor for excessive study behaviour and burnout

The integration of academic self-esteem contingency into the process of burnout in different (professional) contexts is based on both theoretical and empirical evidence [20, 24, 25]. Freudenberger [26] and Farber [25] have already postulated that only highly committed people with burning ambitions are affected by burnout. The integrative model of burnout [27] also postulates that people with high initial motivation and outstanding commitment are more likely to burn out. As high levels of engagement and motivation have generally been considered desirable and do not necessarily lead to exhaustion and cynicism [28], Svedberg and colleagues [25] concluded that this type of motivation must be of a particularly vulnerable nature. Hallsten [19] followed this idea back in 2005 when, in the context of the self-esteem model of burnout, he postulated that people who define their self-esteem primarily in terms of academic achievement are at a greater risk of burning out due to excessive engagement. The initial observations on which Hallsten’s thesis was based were derived from interviews with individuals who were experiencing burnout. These individuals expressed that their self-esteem was contingent upon their achievements, which they perceived to be reflected in the script “I am what I achieve.”

Academic self-esteem contingency describes how self-esteem is formed and maintained [29–31], portraying a self-esteem that is fuelled by the fulfilment of introjected academic expectations [32, 33]. A particular reactivity of self-esteem to (lack of) academic success has been considered a common vulnerability factor (for an overview, see [34]) associated with internalised extrinsic motivations, test anxiety, and stress [35, 36]. When one’s self-esteem is particularly dependent on achievement, the possibility of failure is perceived as a significant threat. Consequently, learning processes (during studies) are accompanied by high levels of tension and anxiety [37–39]. Since students with a high self-esteem contingency tend to make considerable efforts to avoid potential failures [40] and to maintain their self-esteem [41, 42], excessive study behaviour serves them as a form of self-esteem compensation.

In accordance with the assumption that excessive study behaviour serves to compensate for self-esteem (based on a high academic self-esteem contingency) and can ultimately lead to burnout, Hallsten et al. [19] described the initial phase of the burnout process as anxiety-driven engagement, which is characterised by high engagement at the behavioural level and by worries and anxieties at the emotional level. The authors posited that chronic stress, repeated difficulties, coping efforts, and constant worry in the pursuit of high self-esteem ultimately result in efforts that are both exhausting and frustrating, leading to burnout, particularly in the context of setbacks. Accordingly, the authors saw academic self-esteem contingency as the missing piece of the puzzle in providing a plausible explanation for the shift in the burnout process from high engagement to (excessive engagement), strain, stress, and cynicism [6, 18, 27].

Additionally, empirical evidence indicates that engagement driven by an inner compulsion leads to emotional exhaustion and disappointment [43, 44]. Further, excessive study behaviour has shown to be associated with lower academic performance (e.g., [12]), and even individual failures can have a detrimental effect on students with a pronounced self-esteem contingency, reinforcing feelings of reduced self-efficacy. Exhaustion has been shown to reduce an individual’s performance [45], and reduced self-efficacy has shown to be associated with the use of surface-oriented rather than depth-oriented learning strategies [46], making failure even more likely in the long run. When confronted with a multitude of failures (due to excessive engagement), students may ultimately be tempted to devalue their studies and adopt a cynical attitude towards the university in order to protect their own self-esteem.

Consequently, research question 1 of this study examines academic self-esteem contingency as a key vulnerability factor in the development of academic burnout, mediated by excessive study behaviour. Specifically, academic self-esteem contingency is expected to have a positive effect on excessive study behaviour, which in turn is expected to have a positive effect on exhaustion, reduced self-efficacy, and cynicism during studies (Hypothesis 1).

### (Serial) mediation of the effect of parental academic negative conditional regard on academic burnout

The theoretical assumptions of Hallsten et al. [19] and the empirical findings of Svedberg et al. [25] indicate that the common aetiology of academic self-esteem contingency (and excessive study behaviour) and academic burnout is not solely genetic, but also influenced by early socialisation experiences. The family – including parents – as a social reference system can provide resources, but at the same time make demands that can potentially lead to stress [47]. Indeed, there is some empirical evidence that facets of parenting can contribute to academic burnout (cf., [41,48,49]).

Parental academic negative conditional regard is a parenting strategy through which parents withdraw affection, appreciation, and love when their child’s academic performance does not meet their expectations [50, 51], thereby increasing the risk of student burnout [41]. By setting standards for behaviour worthy of approval and rejection, parents (in the terminology of self-determination theory [SDT]; [42,52]) foster ambivalence between the experience of the central basic needs of autonomy and relatedness [53–55], making it impossible to satisfy both basic needs at the same time. Children are thus forced to choose between prioritising their own needs and meeting their parents’ expectations in order to feel related and valued. The long-term negative effects of this parenting strategy (e.g., introjected motivation, self-esteem contingency, and higher risk of depressive symptoms) have already been widely demonstrated (cf., [23]).

Children learn to link their own worth to (external) conditions and to engage in a continuous process of self-validation (cf., [56]) – they develop a self-esteem that is contingent on their academic achievements [57]. In order to avoid the withdrawal of parental love, children tend to introject parental expectations and subsequently orientate their future actions towards them [58, 59]. Those driven by the internalised script “I am what I achieve” and thus constantly striving to protect their own self-esteem pursue their introjected expectations excessively (beyond their own limits) to avoid academic failure at any cost. Thus, the engagement of these children (and later university students), whose self-esteem has been conditional since school age, is based on discipline and control rather than autonomous motivation (e.g., interest; [42]), making task completion more strenuous and exhausting [60, 61].

Although the role of parental conditional regard (mediated by academic self-esteem contingency) in increasing the risk of burnout in the school context has already been well established [41], key questions remain unanswered: Do the effects of parental (negative) conditional regard at school age (in terms of increasing the risk of academic burnout) extend to the university context? Does excessive study behaviour act as a further mediator of the relationship between parental (negative) conditional regard and burnout? Does parental negative conditional regard affect the three burnout dimensions of exhaustion, cynicism, and reduced self-efficacy equally, in a serial manner, via self-esteem contingency and excessive study behaviour?

Recognising that these questions cannot be definitively answered using a cross-sectional design, this study aims to provide a preliminary approach to addressing them. Based on theoretical considerations, it is hypothesised that parental negative conditional regard at school age increases vulnerability to experiencing exhaustion, cynicism, and reduced self-efficacy during studies, serially mediated by academic self-esteem contingency and excessive study behaviour (Hypothesis 2).

### Consideration of covariates in the model

As excessive study behaviour (which is based on discipline and is related to impaired psychosocial functioning) may share common components of time and effort with academic engagement (which is associated with autonomous motivation and higher well-being), Atroszko et al. [2] recommended counteracting the confounding effects by controlling for academic engagement in order to avoid biased results. Furthermore, the prevalence of excessive study behaviour is higher among women than among men (e.g., [2]); this may be due to differences in the way women and men cope with stress [62], with women being more sensitive to punishment [63] and reporting higher average academic self-esteem [64]. To minimise the confounding effects of academic engagement and gender on this study’s findings, these variables were controlled for in this research.

## Method

### Participants and procedure

The final sample consisted of *N* = 624 German university students (age: *M* = 23.63 years, *SD* = 4.16; gender: 73.52% female, 25.36% male, 1.12% diverse). Within this sample, *n* = 71 students had a migrant background. On average, the students were in their fifth semester (*M* = 4.77; *SD* = 2.90), with *n* = 428 students aiming for a bachelor’s degree, *n* = 174 for a master’s degree, and *n* = 22 for a state examination. A total of 39.7% of students were studying psychology, while another 39.4% were studying to become teachers. The remaining 20.9% were enrolled in a range of other academic disciplines. The majority of students (74.78%) worked alongside their studies for varying amounts of time (*M* = 11.74, *SD* = 7.1). Around 6.1% of the mothers and 4.8% of the fathers of the students had no school-leaving qualifications or had completed lower secondary education without a vocational qualification. Conversely, 27.6% of mothers and 34.3% of fathers held a university degree.

At the beginning of the winter semester of 2023/24, students studying teaching and psychology at various German universities were contacted by email to take part in a voluntary online survey. Psychology students at two specific universities were able to receive one credit hour for participating in the study, while no other incentives were offered to students from other disciplines and universities. Approval for the study was obtained from the local ethics committee, and the online survey was conducted in accordance with relevant guidelines and regulations. In addition to the constructs relevant to this study, other variables (such as procrastination, stress mindset, and self-handicapping) were measured as part of the 30-minute online survey through Qualtrics, but were not included in this study. Due to economic reasons, not all constructs were presented to all students; only *n* = 334 students responded to the scale on excessive study behaviour.

Originally, *N* = 702 students took part in the study. However, data of *n* = 71 students were excluded from the study because these students had started their bachelor’s degree in the winter semester and were therefore unable to provide a retrospective account of burnout during their studies. Further, *n* = 7 students were excluded from the analyses because they did not provide information regarding their semester of study.

### Measures

All items were sourced from established measurement instruments and presented in a randomised order. All measurements have been published elsewhere and are cited accordingly.

#### Parental academic negative conditional regard

Parental academic negative conditional regard was measured retrospectively for the time of school age using six items adapted from the German Parental Academic Conditional Regard Inventory [50, 58] and answered on a 5-point Likert scale (1 = *not true at all*, 5 = *exactly right*). In terms of parental negative conditional regard, the instrument records parental withdrawal of attention based on a child’s school performance (e.g., “When I got a bad grade at school, I realised that my primary caregiver was showing me less affection than usual”). A one-factor confirmatory factor analysis (CFA) solution fit the data well (CFI = 0.99; RMSEA = 0.08). Moreover, a reliability analysis showed excellent internal consistency (α = 0.91).

#### Academic self-esteem contingency

Academic self-esteem contingency was assessed using the Self-Esteem Inventory for Children and Adolescents [SEKJ; 33] and rated on a 5-point Likert scale (1 = *not true at all*, 5 = *exactly right*). The original scale of 10 items was shortened to four items and adapted to the university context (e.g. “My self-esteem goes down when others at university get better grades than I do”). The one-factor CFA solution showed an excellent fit (CFI = 0.99; RMSEA = 0.05) and a reliability analysis indicated good internal consistency (α = 0.82).

#### Excessive study behaviour

The German version of the Bergen Study Addiction Scale [BStAS; 7] was used to measure excessive study behaviour. The scale comprises seven items pertaining to the preceding 12 months (e.g., “How often during the last year have you deprioritised hobbies, leisure activities, and exercise because of your studying?”), which are answered on a 5-point Likert scale (1 = *never*, 5 = *always*). The one-factor CFA solution fit the data well (CFI = 0.97; RMSEA = 0.07) after removing item 1 (“How often during the last year have you thought of how you could free up more time to study?”) from the scale due to a very low loading value (< 0.4). Item 1 has already been discussed in the original version in English and in the German validation of the BStAS with regard to low loadings and has been repeatedly removed from the adaptations to other languages [2]. The shortened BStAS showed good internal consistency (α = 0.80).

#### Academic burnout

The German version of the Maslach Burnout Inventory Student Survey Short Form (MBI-SS KV; [65]) uses nine items to assess the burnout dimensions of exhaustion, cynicism, and reduced self-efficacy during studies. Three items corresponding to each of three dimensions were rated on a 5-point Likert scale (1 = *never*, 5 = *very often*). According to the results of the CFA, the three-factor structure fit the data well (CFI = 0.99; RMSEA = 0.04). An example item on the *exhaustion* scale is “I feel burned out from my studies” (α = 0.78). An example item on the *cynicism* scale is “I have become less enthusiastic about my studies” (α = 0.84). Finally, an example item on the *reduced self-efficacy* scale is “In class I don’t feel confident that I can get things done effectively” (α = 0.65).

#### Academic engagement

Academic engagement was measured using six items adapted from the German version of the Utrecht Work Engagement Scale Student Form (UWES-9-SF; [66]). In the adapted scale, the variable was measured using two items from each of the dimensions of vigour (e.g., “When I study, I feel like I am bursting with energy”), absorption (e.g., “Time flies when I’m studying”), and dedication (e.g., “My studies inspire me”). All items were rated on a 5-point Likert scale (1 = *never*, 5 = *very often*). The one-factor CFA solution of higher order fit the data well (CFI = 0.96; RMSEA = 0.08) and a reliability analysis indicated good internal consistency (α = 0.83).

### Analysis strategy

All analyses were conducted in RStudio (version 2023.06.2 + 561; [67]), specifically using the lavaan 0.6–9 package [68]. Maximum likelihood estimator, which is robust to violations of normality assumptions [69], was used for model estimation. To handle missing values (in particular the completely missing at random values of *n* = 282 students on excessive study behaviour), the full information maximum likelihood (FIML) procedure was used. FIML is unbiased under the missing at random (MAR) assumption and retains statistical power as no observations are deleted [70]. Parental academic negative conditional regard, academic self-esteem contingency, excessive study behaviour, academic burnout, and engagement were specified as latent constructs with multiple indicators using effects-coding [71]. For this purpose, the sum of the factor loadings of each construct was set to the number of construct-specific indicators, and the sum of the intercepts of all indicators of one construct was set to 0. Gender was included in the model using two dummy variables for male (1 = *male*; 0 = *not male*) and female (1 = *female*; 0 = *not female*) genders, with diverse as the reference category.

After the latent means and correlations of the relevant variables were checked, the hypothesised structural equation model was tested. In order to identify misspecifications at the level of the measurement model and to distinguish these from misspecifications in the structural model, the “two-step approach” of Herting and Costern (as cited in [72]) was used. This approach involves first calculating a CFA with all measurement models and intercorrelating latent variables in order to estimate the structural model in a further step. The model fit was evaluated using the χ^2^-test and the Comparative Fit Index (CFI), the Tucker Lewis Index (TLI), the Root Mean Square Error of Approximation (RMSEA), and the Standardised Root Mean Square Residual (SRMR). A CFI and TLI above 0.95 combined with an RSMEA and SRMR below 0.06 indicate an excellent fit, while a CFI and TLI above 0.90 and an RMSEA and SRMR below 0.08 indicate an acceptable fit [73]. In the final step, the postulated serial mediation model was specified, based on the methodological paper by Lemardelet and Caron [74], and tested using the bootstrap method. The model included all direct effects and simultaneously all simple and serial mediation effects (as well as effects based on covariates). Each effect was tested for significance while controlling for the other effects.

## Results

### Preliminary analyses

Table 1 shows the latent means, standard deviations, and correlations of all variables examined in the model. Parental negative conditional regard showed a small positive correlation with academic self-esteem contingency, excessive study behaviour, and the burnout dimensions of exhaustion, cynicism, and reduced self-efficacy. Academic self-esteem contingency and excessive study behaviour correlated strongly positive and showed moderate to high correlations with exhaustion and reduced self-efficacy, while correlations with cynicism were smaller. In terms of covariates, academic engagement showed strong negative correlations with the three burnout dimensions, but no significant correlations with parental negative conditional regard, academic self-esteem contingency, and excessive study behaviour. Furthermore, gender correlated small to moderate with academic self-esteem contingency, excessive study behaviour, and exhaustion (with higher values among women than among men).

In consideration of the stress levels exhibited by the present student sample, it was found that 23.42% of the students answered “often” or “always” to at least four of the seven items on the scale of excessive study behaviour, indicating a tendency towards “study addiction” as defined by Atroszko et al. [75]. The proportion was slightly higher than the international prevalence rates of 5.9–20.9% [2], but lower than the rate in the German validation sample (27%; 7), in which no face-to-face teaching took place in 2021 due to the coronavirus. Prevalence rates for burnout vary according to cut-off criteria, and no validated cut-offs are yet available for university students [76]. Some authors posit that sub-dimensions of burnout are present when the corresponding sum index is ≥ 4 [3, 77], indicating frequent occurrence. While other studies have reported much higher rates of exhaustion (24.4%) and cynicism [22.9%; e.g., 3], in the present sample, 10.74% of students experienced symptoms of exhaustion, 6.73% experienced cynicism, and 4.81% experienced reduced self-efficacy “often” to “very often”.

### (Serial) mediation model

The structural equation model showed a good fit: χ^2^_(472)_ = 1035.62, *p* < .001; CFI = 0.92; TLI = 0.92; RMSEA = 0.05, CI [0.04, 0.05]; SRMR = 0.06. It explained a total of 12.4% of the variance in negative self-esteem contingency, 34.6% in excessive study behaviour, 72.6% in exhaustion, 47.2% in cynicism, and 51.2% in reduced self-efficacy. Figure 2 shows all significant direct effects; for the sake of completeness, all (in)direct effects are presented in Appendix 1.

With regard to the first research question, academic self-esteem contingency was found to have a positive effect on all three burnout dimensions. A significant total effect was observed for the relationship between academic self-esteem contingency and exhaustion during studies (β = 0.41, 95% CI [0.32, 0.50]), which proceeded directly (β = 0.15, 95% CI [0.02, 0.28]) and was significantly mediated by excessive study behaviour (β = 0.27, 95% CI [0.17, 0.37]). The effect of academic self-esteem contingency on cynicism was exclusively direct (β = 0.16, 95% CI [0.04, 0.28]), that is, it was not mediated by excessive study behaviour. For reduced self-efficacy, there was also a significant total effect based on academic self-esteem contingency (β = 0.47, 95% CI [0.37, 0.56]), which, on the one hand, proceeded directly (β = 0.37, 95% CI [0.23, 0.51]) and, on the other hand, was mediated by excessive study behaviour (β = 0.09, 95% CI [0.01, 0.18]). Hypothesis 1 was thus partially confirmed, as only a direct effect was found for cynicism.

Regarding the second research question, a significant total effect of parental negative conditional regard on excessive study behaviour was found (β = 0.18, 95% CI [0.07, 0.28]), mediated by academic self-esteem contingency (β = 0.12, 95% CI [0.07, 0.17]). In addition, it was found that parental negative conditional regard at school age increased the risk of exhaustion and cynicism at university, mediated by academic self-esteem contingency (and, for exhaustion, additionally mediated by excessive study behaviour). A significant total effect was observed (β = 0.13, 95% CI [0.04, 0.21]) regarding the relationship between parental negative conditional regard and exhaustion. This consisted of a significant serial mediation effect via academic self-esteem contingency and excessive study behaviour (β = 0.06, 95% CI [0.03, 0.09]), and an additional simple mediation effect, mediated only by academic self-esteem contingency (β = 0.03, 95% CI [0.01, 0.07]). Again, a significant total effect (β = 0.10, 95% CI [0.02, 0.18]) was found, with academic self-esteem contingency (β = 0.04, 95% CI [0.01, 0.07]) acting as a mediator for the relationship between parental negative conditional regard and cynicism. Lastly, there was no significant total effect of parental negative conditional regard at school age on reduced self-efficacy during studies (β = 0.09, 95% CI [-0.01, 19]). Hypothesis 2 was therefore only partially confirmed.

## Discussion

This study addressed two research questions: (1) Is the relationship between academic self-esteem contingency and the individual burnout dimensions of exhaustion, cynicism, and reduced self-efficacy mediated by excessive study behaviour? And (2) does parental negative conditional regard during school age increase vulnerability to experiencing exhaustion, cynicism, and reduced self-efficacy during study, mediated by academic self-esteem contingency and excessive study behaviour? These research questions were examined using a serial mediation model, while controlling for academic engagement and gender, in a sample of 624 students.

### Mediation of the relationship between academic Self-Esteem contingency and burnout via excessive study behaviour

With regard to the first research question and in conformity with Hypothesis 1, the findings highlight that students with a self-esteem dependent on academic performance were more likely to develop burnout symptoms in terms of exhaustion, cynicism, and reduced self-efficacy. Furthermore, students with a high academic self-esteem contingency were more likely to show over-involvement in their studies, which partially mediated the effect on the burnout dimensions of exhaustion and reduced self-efficacy. These findings support Hallsten’s [19] self-esteem model of burnout, in which performance-based self-esteem is postulated as a significant motivator of a particularly vulnerable nature, driving excessive engagement that leads to burnout (especially exhaustion) in the long run.

Students with a highly contingent academic self-esteem often engage in excessive study behaviour in order to actively avoid failure and the associated feelings of shame and guilt, thus protecting their self-esteem. As part of their excessive study behaviour, students are increasingly prone to overstretch and push themselves beyond their limits. Due to the associated lack of resource recovery (leisure activities and rest periods), they do not seem to be able to meet (their own) performance aspirations during their studies in the long run. As a result, students burn out – suffering from exhaustion and reduced self-efficacy.

This result is consistent with the current state of research, as studies have already demonstrated that academic self-esteem contingency is associated with burnout (e.g., [24]) and that excessive study behaviour is a significant predictor of academic burnout (e.g., [6]). The findings of this study thus complement the picture by providing empirical evidence of the mediating role of excessive study behaviour for the effect of academic self-esteem contingency on exhaustion and reduced self-efficacy.

Contrary to Hypothesis 1, however, the effect of academic self-esteem contingency on cynicism during studies was found to be exclusively direct; this relationship was not mediated by excessive study behaviour. This finding seems plausible insofar as excessive study behaviour implies that a student’s current lifestyle is largely oriented towards studying, for instance, with leisure activities put on hold (cf., [7]). According to this understanding, a cynical attitude towards studying would imply a loss of the foundation of one’s personal identity and self-esteem. It is possible that students whose self-esteem depends on (lack of) academic success tend to protect their own worth either actively (through excessive study behaviour) or passively (through devaluation and a cynical attitude towards their studies). Rather than exerting excessive effort to actively avoid failure, some students may alter their evaluation of the study programme, thus perceiving failure as less threatening to their self-esteem. One possible reason for choosing this coping mechanism could be a fixed mindset, which is defined as the belief that one’s intelligence or performance is unchangeable by effort (cf., [78]).

### (Serial) mediation of the relationship between parental negative conditional regard and burnout via academic self-esteem contingency and excessive study behaviour

With regard to the second research question, and in line with Hypothesis 2 as well as current research [23, 56], this study’s findings show that the self-esteem of students whose parents reacted to their failure to meet academic expectations at school by withdrawing appreciation was more likely to depend on academic performance. Through academic negative conditional regard at school age, parents (unwittingly) increase their children’s vulnerability to long-term, unhealthy, and excessive study behaviour, which serves as self-esteem protection.

Complementing recent research by Lavrijsen and colleagues [41], who demonstrated an (indirect) effect of parental conditional regard on burnout in the school context, the results of the present study suggest that parental negative conditional regard at school age increases vulnerability to experiencing exhaustion and cynicism in the long run (i.e. also at university) through promoting academic self-esteem contingency. Excessive study behaviour acted as an additional mediator of the effect of parental negative conditional regard at school age on exhaustion, but not on cynicism, at university. However, an (in)direct effect of parental negative conditional regard at school age on reduced academic self-efficacy at university could not be found, so Hypothesis 2 was only partially confirmed.

According to the findings of this study, parental negative conditional regard during school years has a long-term positive effect on exhaustion during university, the key symptom of burnout [79–81]. On the one hand, parental academic negative conditional regard at school may already increase the likelihood of (emotional) exhaustion during studies through increasing the dependence of a child’s self-esteem on (lack of) academic success and the associated threat to their self-esteem: Learning situations have shown to be associated with increased tension and anxiety [37, 39], and the pressure and stress experienced internally can lead directly to (emotional) exhaustion and academic burnout [82]. On the other hand, these students are more likely to engage in excessive study behaviour in order to avoid feelings of worthlessness, which also increases their likelihood of experiencing exhaustion.

The effect of parental negative conditional regard at school on cynicism at university was exclusively mediated by academic self-esteem contingency, but not by excessive study behaviour. This outcome is consistent with previous findings that parental negative conditional regard during school years is conducive to the development of an increased self-esteem contingency in children (e.g., [23]). However, parental negative conditional regard has also shown to be associated with reactance or amotivation at school [56], which tends to form a counterbalance to excessive study behaviour. In line with previous research [83], the findings of this study can be interpreted as indicating that cynicism represents an alternative form of self-esteem protection (in contrast to excessive study behaviour) and is also caused by parental negative conditional regard. Regarding the frequency of use of these differentiated coping strategies, the small effect of academic self-esteem contingency on cynicism compared to its large effect on excessive study behaviour could be interpreted as an indication that a self-esteem dependent on academic performance leads students to protect their self-esteem more often (actively) through excessive study behaviour and less often (passively) through a cynical attitude towards their studies. The extent to which this is actually the case and the moderators [78] that explain the use of differentiated coping mechanisms in response to an academically contingent self-esteem require further research.

According to the results of this study, parental negative conditional regard during the school years has no (indirect) effect on experiencing reduced self-efficacy at university. One possible explanation could be that meeting parental expectations is associated with the experience of self-efficacy among pupils, but not among university students: Given that every parent has been to school, it can be assumed that parents know what they are talking about. Nevertheless, this principle may not apply to the study programme as a subject-specific qualification. Even if the parents have a university degree, they are often trained in a completely different field, and the requirements are less transferable. From the students’ point of view, parents may therefore be less able to assess their competences during their studies. Consequently, parents’ expectations and attributions of competence become less important at university. The findings of a study conducted by Lavrijsen and colleagues [41] in the school context provide empirical support for this idea; in this study, a greater influence on the development of burnout symptoms was found for teachers’ conditional regard than for parents’. The authors explained this effect by suggesting that teachers, because of their proximity to the context, serve as the most important source of feedback for assessing pupils’ performance. This idea may apply equally to lecturers and students, but requires further research.

In conclusion, parental negative conditional regard during school years can have long-term negative health effects for children, which can persist until university. However, the results of this study indicate that parental negative conditional regard does not affect all three burnout dimensions equally. Although academic self-esteem contingency, fostered by parental negative conditional regard, was a vulnerability factor for the development of exhaustion and cynicism (at university), the mechanisms of action appeared to be different. Thus, excessive study behaviour acted as another mediator of the effect of parental negative conditional regard on exhaustion, but not on cynicism.

### Limitations

In addition to the strengths of the study (e.g., large sample size, sophisticated analysis method), it is important to note some of its limitations. First, the serial meditation model was tested using a cross-sectional design and was based entirely on self-reported data, which implies that causal interpretations are inadmissible. In a strict sense, it is possible to speak not of effects, but only of correlations. Furthermore, a judgemental bias cannot be ruled out. Consequently, further research is required to test the reported results in a longitudinal design and to consider objective measures to assess excessive study behaviour or academic self-esteem contingency. Second, the sample is rather selective, comprising primarily students of psychology and teaching from two specific universities. Hence, future research should examine the generalisability of the findings to other student populations. Third, academic burnout is influenced by a variety of environmental factors, such as teacher/lecturer-student relationships [84] or peer support [85], which were not considered in this study. Future research should examine whether different environmental factors are likely to attenuate or amplify the relationship between parenting, personality, and the subsequent development of excessive engagement and burnout, in order to provide a more comprehensive understanding of burnout development.

### Practical implications

According to the findings of this study, academic self-esteem contingency is a key vulnerability factor for the development of excessive study behaviour and academic burnout. This finding suggests that in order to prevent and intervene in excessive study behaviour and academic burnout, it is not enough to work only at the behavioural level through activating resources and planning time for breaks and relaxation. Rather, students need to reflect on how they evaluate their own failures and what such evaluation means for their own self-esteem. They also need to question their own internalised demands and learn to deal with feelings of shame and guilt that arise when prioritising non-academic activities (such as hobbies). Only in this way will it be possible for them to regain the freedom to fully enjoy their student life by actively planning time for leisure activities, friends, and self-care, and thus to change their behaviour in a health-promoting way – not least to counteract the chronification of an unhealthy work and lifestyle.

## Conclusion

The findings of this study suggest that by inducing feelings of shame and guilt, and through promoting the contingency of self-esteem on academic performance, parental negative academic conditional regard at school age contributes to students’ long-term prioritisation of their academic careers over leisure and health. This effect may initially appear beneficial in the eyes of parents, who may perceive their children’s decision to invest considerable effort in their professional future as a positive indicator. Nevertheless, the underlying compulsive and addiction-like mechanisms, the high levels of stress, and the associated (emotional) exhaustion are observable only over a longer period of time and often lead to individuals dropping out of university or work. Thus, this study provides evidence that not only is the risk of academic burnout increased by current structural and contextual factors, but also family socialisation has an impact on increasing a student’s vulnerability to academic burnout. Consequently, there is a need for preventive measures (e.g., psychoeducation) to reduce the risk of burnout in (young) adulthood, thus counteracting the long-term health and financial costs to individuals and society.

## Supplementary Information

Below is the link to the electronic supplementary material.

Supplementary Material 1

## Data Availability

The datasets generated during and/or analysed during the current study are not publicly available [as there is no consent of the participants for the general publication of the data] but datasets are available from the corresponding author on reasonable request.

## Abbreviations

BStAS: Bergen study addiction scale
CFA: Confirmatory factor analysis
CFI: Comparative fit index
CI: Confidence interval
FIML: Full information maximum likelihood
MAR: Missing at random
MBI-SS KV: Maslach burnout inventory student survey short form
RMSEA: Root mean square error of approximation
SDT: Self-determination theory
SEKJ: Self-esteem inventory for children and adolescents
SRMR: Standardised root mean square residual
TLI: Tucker-Lewis index
UWES-9-SF: Utrecht work engagement scale student form

## References

[1] Atroszko PA, Sawicki A, Kamble S. Cross-cultural pilot study on the relationship between study addiction and narcissism among undergraduate students in Poland and India. Health Psychol Rep. 2019;7(4):325–33.

[2] Atroszko PA, Charzyńska E, Buźniak A, Czerwiński SK, Griffiths MD, Jankowska A et al. Validity, reliability, and cross-cultural comparability of a problematic overstudying scale across european, North american, and Asian countries. Int J Ment Health Addict. 2023;1–23.

[3] Grützmacher J, Gusy B, Lesener T, Sudheimer S, Willige J. Gesundheit Studierender in Deutschland 2017 [Student health in Germany 2017]. Ein Kooperationsprojekt zwischen dem Deutschen Zentrum für Hochschul- und Wissenschaftsforschung, der Freien Universität Berlin und der Techniker Krankenkasse.; 2018 [cited 2024 May 15]. Available from: https://www.dzhw.eu/pdf/21/gesundheit_studierender_in_deutschland_2017.pdf

[4] Söderholm F, Lappalainen K, Holopainen L, Viljaranta J. The development of school burnout in general upper secondary education: the role of support and schoolwork difficulties. Educ Psychol. 2022;42(5):607–25.

[5] Cilliers JR, Mostert K, Nel JA. Study demands, study resources and the role of personality characteristics in predicting the engagement of first-year university students. South Afr J High Educ. 2018;32(1):49–70.

[6] Bereznowski P, Atroszko PA, Konarski R. Work addiction, work engagement, job burnout, and perceived stress: A network analysis. Front Psychol. 2023;14:1130069. 37063548 10.3389/fpsyg.2023.1130069 PMC10090512

[7] Schaefer J, Strob J. Wenn Das studieren Außer kontrolle gerät: entwicklung und validierung einer deutschsprachigen adaptation der Bergen study addiction scale (BStAS) [When studying gets out of control: Development and validation of a german adaptation of the bergen study addiction scale]. Z Für Klin Psychol Psychother. 2023;52(1):25–37.

[8] Sun R, Yang HM, Chau CTJ, Cheong IS, Wu AMS. Psychological empowerment, work addiction, and burnout among mental health professionals. Curr Psychol. 2023;42(29):25602–13.

[9] Woropay-Hordziejewicz NA, Buźniak A, Lawendowski R, Atroszko PA. Compulsive study behaviors are associated with eating disorders and have independent negative effects on well-being: A structural equation model study among young musicians. Sustainability. 2022;14(14):8617.

[10] Schaufeli WB, Martínez IM, Pinto AM, Salanova M, Bakker AB. Burnout and engagement in university students: A cross-national study. J Cross-Cult Psychol. 2002;33(5):464–81.

[11] Walburg V. Burnout among high school students: A literature review. Child Youth Serv Rev. 2014;42:28–33.

[12] Lawendowski R, Bereznowski P, Wróbel WK, Kierzkowski M, Atroszko PA. Study addiction among musicians: measurement, and relationship with personality, social anxiety, performance, and psychosocial functioning. Music Sci. 2020;24(4):449–74.

[13] Mostert K, Pienaar J. The moderating effect of social support on the relationship between burnout, intention to drop out, and satisfaction with studies of first-year university students. J Psychol Afr. 2020;30(3):197–202.

[14] Peng P, Chen S, Hao Y, He L, Wang Q, Zhou Y, et al. Network of burnout, depression, anxiety, and dropout intention in medical undergraduates. Int J Soc Psychiatry. 2023;69(6):1520–31. 37092762 10.1177/00207640231166629

[15] Andreassen CS, Griffiths MD, Hetland J, Pallesen S. Development of a work addiction scale. Scand J Psychol. 2012;53(3):265–72. 22490005 10.1111/j.1467-9450.2012.00947.x

[16] Atroszko PA, Andreassen CS, Griffiths MD, Pallesen S. The relationship between study addiction and work addiction: A cross-cultural longitudinal study. J Behav Addict. 2016;5(4):708–14. 27842448 10.1556/2006.5.2016.076 PMC5370377

[17] Griffiths MD, Demetrovics Z, Atroszko PA. Ten Myths about work addiction. J Behav Addict. 2018;7(4):845–57. 29409339 10.1556/2006.7.2018.05 PMC6376361

[18] Bereznowski P, Konarski R, Pallesen S, Atroszko PA. Similarities and differences between study addiction and study engagement and work addiction and work engagement: A network analysis. Int J Ment Health Addict. 2024;1–22.

[19] Hallsten L. Burnout and wornout - concepts and data from a National survey. In: Antoniou ASG, Cooper CL, editors. Research companion to organizational health psychology. Edwad Elgar Publishing; 2005. pp. 516–36.

[20] Dahlin M, Joneborg N, Runeson B. Performance-based self-esteem and burnout in a cross-sectional study of medical students. Med Teach. 2009;29(1):43–8. 10.1080/01421590601175309 17538833

[21] Love H, May RW, Cui M, Fincham FD. Helicopter parenting, self-control, and school burnout among emerging adults. J Child Fam Stud. 2020;29(2):327–37.

[22] Salmela-Aro K, Upadyaya K. School burnout and engagement in the context of demands–resources model. Br J Educ Psychol. 2014;84(1):137–51. 24547758 10.1111/bjep.12018

[23] Haines JE, Schutte NS. Parental conditional regard: A meta-analysis. J Adolesc. 2023;95(2):195–223. 36345118 10.1002/jad.12111

[24] Blom V. Contingent self-esteem, stressors and burnout in working women and men. Work. 2012;43(2):123–31. 22927616 10.3233/WOR-2012-1366

[25] Svedberg P, Hallsten L, Narusyte J, Bodin L, Blom V. Genetic and environmental influences on the association between performance-based self-esteem and exhaustion: A study of the self-worth notion of burnout. Scand J Psychol. 2016;57(5):419–26. 27452914 10.1111/sjop.12309

[26] Freudenberger HJ. Staff burnout. J Soc Issues. 1974;30(1):159–65.

[27] Schaufeli W, Enzmann D. The burnout companion to study and practice: A critical analysis. London: CRC; 1998.

[28] Schmitz E, Leidl J. Brennt Wirklich aus, Wer Entflammt war? Studie 2: eine LISREL-Analyse zum Burnout-Prozess Bei lehrpersonen [Do people really burn out, who Were once enflamed? Study 2: A LISREL analysis of burnout process in teachers]. Psychol Erzieh Unterr. 1999;46(4):302–10.

[29] Crocker J, Wolfe CT. Contingencies of self-worth. Psychol Rev. 2001;108(3):593–623. 11488379 10.1037/0033-295x.108.3.593

[30] Crocker J, Park LE. The costly pursuit of self-esteem. Psychol Bull. 2004;130(3):392–414. 15122925 10.1037/0033-2909.130.3.392

[31] Kernis MH. Toward a conceptualization of optimal self-esteem. Psychol Inq. 2003;14(1):1–26.

[32] Otterpohl N, Bruch S, Stiensmeier-Pelster J, Steffgen T, Schöne C, Schwinger M. Clarifying the connection between parental conditional regard and contingent self-esteem: an examination of cross-lagged relations in early adolescence. J Pers. 2021;89(5):986–97. 33646604 10.1111/jopy.12631

[33] Schöne C, Stiensmeier-Pelster J. SEKJ - Selbstwertinventar für kinder und Jugendliche [Self-esteem inventory for children and adolescents]. Göttingen: Hogrefe; 2016.

[34] Fairlamb S. We need to talk about self-esteem: the effect of contingent self-worth on student achievement and well-being. Scholarsh Teach Learn Psychol. 2022;8(1):45–57.

[35] Lawrence JS, Gonzales JE, Sutherland KT. Academically-contingent self-worth: dimensionality and associations with negative affectivity and achievement goals. Personal Individ Differ. 2021;180:110987.

[36] Lawrence JS, Gonzales JE. Academically-contingent self-worth: different dimensions differentially predict future vulnerability. Curr Psychol. 2023;42(28):24947–61.

[37] Ching BHH, Wu HX, Chen TT. Maternal achievement-oriented psychological control: implications for adolescent academic contingent self-esteem and mathematics anxiety. Int J Behav Dev. 2021;45(3):193–203.

[38] Lawrence JS, Williams A. Anxiety explains why people with domain-contingent self-worth underperform on ability-diagnostic tests. J Res Personal. 2013;47(3):227–32.

[39] van der Kaap-Deeder J, Wouters S, Verschueren K, Briers V, Deeren B, Vansteenkiste M. The pursuit of self-esteem and its motivational implications. Psychol Belg. 2016;56(3):143–68. 30479434 10.5334/pb.277 PMC5854109

[40] Osborne JW, Jones BD. Identification with academics and motivation to achieve in school: how the structure of the self influences academic outcomes. Educ Psychol Rev. 2011;23:131–58.

[41] Lavrijsen J, Soenens B, Vansteenkiste M, Verschueren K. When insecure self-worth drains students’ energy: academic contingent self-esteem and parents’ and teachers’ perceived conditional regard as predictors of school burnout. J Youth Adolesc. 2023;52(4):810–25. 36807227 10.1007/s10964-023-01749-y

[42] Ryan RM, Deci EL. Self-determination theory and the facilitation of intrinsic motivation, social development, and well-being. Am Psychol. 2000;55(1):68–78. 11392867 10.1037//0003-066x.55.1.68

[43] Garn AC, Jolly JL. A model of parental achievement-oriented psychological control in academically gifted students. High Abil Stud. 2015;26(1):105–16.

[44] Itzhaki-Braun Y, Itzhaky H, Yablon YB. Predictors of high-school dropout among ultraorthodox Jewish youth. Front Psychol. 2020;11:549388. 10.3389/fpsyg.2020.01911 PMC7431703 32849120

[45] Feuerhahn N, Stamov-Roßnagel C, Wolfram M, Bellingrath S, Kudielka BM. Emotional exhaustion and cognitive performance in apparently healthy teachers: A longitudinal multi‐source study. Stress Health. 2013;29(4):297–306. 23086898 10.1002/smi.2467

[46] Prat-Sala M, Redford P. The interplay between motivation, self-efficacy, and approaches to studying. Br J Educ Psychol. 2010;80(2):283–305. 20021729 10.1348/000709909X480563

[47] Salmela-Aro K, Tang X, Upadyaya K. Study demands-resources model of student engagement and burnout. In: Reschly AL, Christenson SL, editors. Handbook of research on student engagement. Cham: Springer International Publishing; 2022. pp. 77–93.

[48] Ching BHH, Li YH, Chen TT. Helicopter parenting contributes to school burnout via self-Control in late adolescence: A longitudinal study. Curr Psychol. 2022;42(33):29699–711.

[49] Raufelder D, Hoferichter F, Ringeisen T, Regner N, Jacke C. The perceived role of parental support and pressure in the interplay of test anxiety and school engagement among adolescents: evidence for gender-specific relations. J Child Fam Stud. 2015;24(12):3742–56.

[50] Roth G, Assor A, Niemiec CP, Ryan RM, Deci EL. The emotional and academic consequences of parental conditional regard: comparing conditional positive regard, conditional negative regard, and autonomy support as parenting practices. Dev Psychol. 2009;45(4):1119–42. 19586183 10.1037/a0015272

[51] Steffgen ST, Soenens B, Otterpohl N, Stiensmeier-Pelster J, Schwinger M. Latent profiles of parental academic conditional positive and negative regard. Parenting. 2022;22(4):347–81.

[52] Ryan RM, editor. The Oxford handbook of self-determination theory. Oxford University Press; 2023.

[53] Cohen R, Moed A, Shoshani A, Roth G, Kanat-Maymon Y. Teachers’ conditional regard and students’ need satisfaction and agentic engagement: A multilevel motivation mediation model. J Youth Adolesc. 2020;49(4):790–803. 31482514 10.1007/s10964-019-01114-y

[54] Kanat-Maymon Y, Roth G, Assor A, Raizer A. Controlled by love: the harmful relational consequences of perceived conditional positive regard. J Pers. 2016;84(4):446–60. 25773317 10.1111/jopy.12171

[55] Moller AC, Roth G, Niemiec CP, Kanat-Maymon Y, Deci EL. Mediators of the associations between parents’ conditional regard and the quality of their adult-children’s peer-relationships. Motiv Emot. 2019;43(1):35–51.

[56] Kanat-Maymon Y, Assor A, Roth G. Conditional regard in development and relationships. In: Ryan RM, editor. The Oxford handbook of Self-Determination theory. 1st ed. Oxford: Oxford University Press; 2023. pp. 548–70.

[57] Otterpohl N, Steffgen ST, Stiensmeier-Pelster J, Brenning K, Soenens B. The intergenerational continuity of parental conditional regard and its role in mothers’ and adolescents’ contingent self-esteem and depressive symptoms. Soc Dev. 2020;29(1):143–58.

[58] Assor A, Roth G, Deci EL. The emotional costs of parents’ conditional regard: A self-determination theory analysis. J Pers. 2004;72(1):47–88. 14686884 10.1111/j.0022-3506.2004.00256.x

[59] Ryan RM, Deci EL. Self determination theory: basic psychological needs in motivation, development, and wellness. Guilford; 2017.

[60] Assor A, Vansteenkiste M, Kaplan A. Identified versus introjected approach and introjected avoidance motivations in school and in sports: the limited benefits of self-worth strivings. J Educ Psychol. 2009;101(2):482–97.

[61] Vansteenkiste M, Smeets S, Soenens B, Lens W, Matos L, Deci EL. Autonomous and controlled regulation of performance-approach goals: their relations to perfectionism and educational outcomes. Motiv Emot. 2010;34(4):333–53.

[62] Brougham RR, Zail CM, Mendoza CM, Miller JR. Stress, sex differences, and coping strategies among college students. Curr Psychol. 2009;28(2):85–97.

[63] Cross CP, Copping LT, Campbell A. Sex differences in impulsivity: A meta-analysis. Psychol Bull. 2011;137(1):97–130. 21219058 10.1037/a0021591

[64] Herrmann J, Koeppen K, Kessels U. Do girls take school too seriously? Investigating gender differences in school burnout from a self-worth perspective. Learn Individ Differ. 2019;69:150–61.

[65] Wörfel F, Gusy B, Lohman K, Kleiber D. Validierung der Deutschen Kurzversion des Maslach-Burnout-Inventars für studierende [Validation of the German short version of the Maslach-Burnout-Inventory for students]. Z Für Gesundheitspsychologie. 2015;23(4):191–6.

[66] Gusy B, Lesener T, Wolter C. Measuring well-being with the Utrecht work engagement Scale – Student form. Eur J Health Psychol. 2019;26(2):31–8.

[67] RStudio Team. RStudio: Integrated Development for R and RStudio. Boston, MA. 2020. Available from: http://www.rstudio.com/

[68] Rosseel Y. lavaan: An R package for Structural Equation Modeling. J Stat Softw. J Stat Softw. 2012;48(2):1–36. Available from: http://www.jstatsoft.org/v48/i02/

[69] Yuan KH, Bentler PM. Three likelihood-based methods for mean and covariance structure analysis with nonnormal missing data. Sociol Methodol. 2000;30(1):165–200.

[70] Enders CK. Applied missing data analysis. 2nd ed. New York: Guilford; 2022.

[71] Little TD, Slegers DW, Card NA. A non-arbitrary method of identifying and scaling latent variables in SEM and MACS models. Struct Equ Model Multidiscip J. 2006;13(1):59–72.

[72] Urban D, Mayerl J, Strukturgleichungsmodellierung. Ein ratgeber für die praxis [Structural equation modelling: A practical guide]. Berlin: Springer; 2014.

[73] Hoyle RH, Panter AT. Writing about structural equation models. In: Hoyle RH, editor. Structural equation modeling: concepts, issues, and applications. Thousand Oaks: Sage; 1995. pp. 158–76.

[74] Lemardelet L, Caron PO, Mplus. R Quant Methods Psychol. 2022;18(1):66–90.

[75] Atroszko PA, Atroszko B, Charzyńska E. Subpopulations of addictive behaviors in different sample types and their relationships with gender, personality, and well-being: latent profile vs. latent class analysis. Int J Environ Res Public Health. 2021;18(16):8590. 34444338 10.3390/ijerph18168590 PMC8394473

[76] Lutz-Kopp C, Meinhardt-Injac B, Luka-Krausgrill U. Psychische belastung studierender [Psychological distress among university students]. Prävent Gesundheitsförderung. 2019;14(3):256–63.

[77] Rolle C, Götte P, Rotthoff T. Gesundheitsförderung auf dem Campus – Wie Es studierenden geht und was Sie Sich Wünschen [Health promotion on campus - how students are doing and what they wish for]. Prävent Gesundheitsförderung. 2024;19(2):286–96. 10.1007/s11553-023-01051-6 PMC10243258 40479178

[78] Altikulaç S, Janssen TWP, Yu J, Nieuwenhuis S, Van Atteveldt NM. Mindset profiles of secondary school students: Associations with academic achievement, motivation and school burnout symptoms. Br J Educ Psychol. 2024;bjep.12676. 10.1111/bjep.12676 38453165

[79] Kristensen TS, Borritz M, Villadsen E, Christensen KB. The Copenhagen burnout inventory: A new tool for the assessment of burnout. Work Stress. 2005;19(3):192–207.

[80] Maslach C, Schaufeli WB, Leiter MP. Job burnout. Annu Rev Psychol. 2001;52(1):397–422. 11148311 10.1146/annurev.psych.52.1.397

[81] Shirom A, Melamed S. A comparison of the construct validity of two burnout measures in two groups of professionals. Int J Stress Manag. 2006;13(2):176–200.

[82] Hill AP, Curran T. Multidimensional perfectionism and burnout: A Meta-Analysis. Personal Soc Psychol Rev. 2016;20(3):269–88. 10.1177/1088868315596286 26231736

[83] Naus F, van Iterson A, Roe RA. Value incongruence, job autonomy, and organization-based self-esteem: A self-based perspective on organizational cynicism. Eur J Work Organ Psychol. 2007;16(2):195–219.

[84] Lee T, Hong SE, Kang J, Lee SM. Role of achievement value, teachers’ autonomy support, and teachers’ academic pressure in promoting academic engagement among high school seniors. Sch Psychol Int. 2023;44(6):629–48.

[85] Lee M, Lee T, Lee SM. Role of peer support in competitive classroom climates: focusing on the mediation effect of academic hatred in the JD-R model. J Psychol Couns Sch. 2023;33(2):221–32.

