# Supplementary material for: Planting the seed: how parental negative conditional regard boosts vulnerability to excessive study behaviour and burnout
Source: BMC Psychol. 2025 Aug 28;13:977. doi: 10.1186/s40359-025-03354-z (PMC12392595; doi:10.1186/s40359-025-03354-z)
Supplement: Supplementary file 1 — Supplementary Material 1 [file 40359_2025_3354_MOESM1_ESM.docx]

**Appendices**

**Appendix 1**

*All Possible (In)direct Effects Within the Model*

|  | *b* | β | *S.E.* | *Z* | CI below | CI above | *p* |
| --- | --- | --- | --- | --- | --- | --- | --- |
| *Parental negative conditional regard* |  |  |  |  |  |  |  |
| **🡪 self-esteem contingency** | **.25** | **.23** | **.04** | **5.86** | **.23** | **.32** | **<.001** |
| 🡪 excessive study engagement | .46 | .06 | .06 | .94 | -.06 | .17 | .35 |
| 🡪 exhaustion | .00 | .00 | .05 | .08 | -.09 | .1 | .94 |
| 🡪 cynicism | .05 | .05 | .04 | 1.13 | -.04 | .13 | .26 |
| 🡪 reduced self-efficacy | -.02 | -.03 | .05 | -.52 | -.12 | .07 | .60 |
| *Self-esteem contingency* |  |  |  |  |  |  |  |
| **🡪 excessive study engagement** | **.41** | **.54** | **.06** | **9.25** | **.42** | **.65** | **<.001** |
| **🡪 exhaustion** | **.12** | **.15** | **.07** | **2.23** | **.02** | **.28** | **.03** |
| **🡪 cynicism** | **.16** | **.16** | **.06** | **2.53** | **.04** | **.28** | **.01** |
| **🡪 reduced self-efficacy** | **.27** | **.37** | **.07** | **5.2** | **.23** | **.51** | **<.001** |
| *Excessive study behaviour* |  |  |  |  |  |  |  |
| **🡪 exhaustion** | **.54** | **.50** | **.07** | **7.16** | **.36** | **.63** | **<.001** |
| 🡪 cynicism | .10 | .08 | .08 | .99 | -.07 | .22 | .32 |
| **🡪 reduced self-efficacy** | **.17** | **.18** | **.08** | **2.12** | **.01** | **.34** | **.03** |
| *Academic engagement* |  |  |  |  |  |  |  |
| 🡪 self-esteem contingency | -.00 | -.00 | .05 | -.03 | -.1 | .09 | .98 |
| 🡪 excessive study behaviour | .08 | .07 | .06 | **1.12** | -.05 | .19 | .26 |
| **🡪 exhaustion** | **-.77** | **-.65** | **.04** | **-15.95** | **-.74** | **-.57** | **<.001** |
| **🡪 cynicism** | **-.92** | **-.65** | **.04** | **-18.78** | **-.72** | **-.58** | **<.001** |
| **🡪 reduced self-efficacy** | **-.56** | **-.54** | **.05** | **-11.46** | **-.64** | **-.45** | **<.001** |
| *Female gender* |  |  |  |  |  |  |  |
| 🡪 self-esteem contingency | -.18 | .09 | .14 | -.62 | -.36 | .19 | .54 |
| 🡪 excessive study behaviour | .39 | -.24 | .18 | 1.34 | -.11 | .59 | .18 |
| 🡪 exhaustion | -.03 | -.02 | .13 | -.12 | -.27 | .24 | .90 |
| 🡪 cynicism | -.34 | -.16 | .14 | -1.13 | -.44 | .12 | .26 |
| 🡪 reduced self-efficacy | -.32 | -.21 | .21 | -.97 | -.63 | .21 | .33 |
| *Male gender* |  |  |  |  |  |  |  |
| **🡪 self-esteem contingency** | **-.76** | **-.35** | **.14** | **-2.48** | **-.63** | **-.07** | **.01** |
| 🡪 excessive study behaviour | .23 | .14 | .18 | .77 | -.22 | .49 | .44 |
| 🡪 exhaustion | .03 | .02 | .13 | .13 | -.24 | .28 | .90 |
| 🡪 cynicism | -.11 | -.05 | .14 | -.37 | -.34 | .23 | .71 |
| 🡪 reduced self-efficacy | -.24 | -.15 | .21 | -.73 | -.57 | .26 | .47 |
|  | | | | | | | |
| *Indirect effects of parental negative conditional regard* | | | | | | | |
| **🡪 self-esteem contingency 🡪 exhaustion** | **.03** | **.03** | **.02** | **2.11** | **.00** | **.07** | **.04** |
| 🡪 excessive study behaviour 🡪 exhaustion | .02 | .03 | .03 | .94 | -.03 | .08 | .35 |
| **🡪 self-esteem contingency 🡪 excessive study behaviour 🡪 exhaustion** | **.06** | **.06** | **.02** | **3.77** | **.03** | **.09** | **<.001** |
| **🡪 self-esteem contingency 🡪 cynicism** | **.04** | **.04** | **.02** | **2.29** | **.01** | **.07** | **.02** |
| 🡪 excessive study behaviour 🡪 cynicism | .00 | .00 | .01 | .68 | -.01 | .02 | .49 |
| 🡪 self-esteem contingency🡪 excessive study behaviour 🡪 cynicism | .01 | .01 | .01 | .96 | -.01 | .03 | .34 |
| **🡪 self-esteem contingency 🡪 reduced self-efficacy** | **.07** | **.08** | **.02** | **3.96** | **.04** | **.13** | **<.001** |
| 🡪 excessive study behaviour 🡪 reduced self-efficacy | .01 | .01 | .01 | .84 | -.01 | .03 | .40 |
| 🡪 self-esteem contingency 🡪 excessive study behaviour 🡪 reduced self-efficacy | .02 | .02 | .01 | 1.89 | -.00 | .04 | .06 |
| **🡪 self-esteem contingency 🡪 excessive study behaviour** | **.10** | **.12** | **.03** | **4.73** | **.07** | **.17** | **<.001** |
| *Indirect effects of academic self-esteem contingency* | | | | | | | |
| **🡪 excessive study behaviour 🡪 exhaustion** | **.22** | **.27** | **.03** | **4.73** | **.07** | **.17** | **<.001** |
| 🡪 excessive study behaviour 🡪 cynicism | .04 | .04 | .04 | .98 | -.04 | .12 | .33 |
| **🡪 excessive study behaviour 🡪 reduced self-efficacy** | **.07** | **.09** | **.05** | **2.06** | **.01** | **.18** | **.04** |

*Note.*  Significant effects in bold.

* *p* < .05. ** *p* < .01. *** *p* < .001.
